# Metal Glycerolate‐Based Synthesis and Structure–Property Relationships of LiNi_1−*x*−*y*_Co_*x*_Mn_*y*_O_2_ Cathodes for Lithium‐Ion Batteries

**DOI:** 10.1002/open.70327

**Published:** 2026-10-28

**Authors:** Gustavo Thalmer M. Silva, Syra Mubarac, Irlan S. Lima, Bruno Freitas, João Pedro A. dos Santos, Josué M. Gonçalves, Hudson Zanin

**Affiliations:** ^1^ Center for Studies in Energy and Oil University of Campinas Campinas Brazil; ^2^ Mackenzie Institute for Research in Graphene and Nanotechnologies (MackGraphe) Mackenzie Presbyterian Institute São Paulo Brazil; ^3^ Advanced Energy Storage Division Center for Innovation on New Energies School of Electrical and Computer Engineering University of Campinas Campinas Brazil; ^4^ Department of Fundamental Chemistry Institute of Chemistry University of São Paulo São Paulo Brazil; ^5^ Mackenzie School of Engineering Mackenzie Presbyterian University São Paulo Brazil

**Keywords:** glycerolate, layered transition oxide metals, lithium‐ion battery, NCM cathode

## Abstract

Lithium‐ion batteries are currently the dominant energy storage systems, with cathode materials playing a significant role in electrochemical performance, safety, and cost. Among these materials, layered LiNi_1−*x*−*y*_Co*_x_* Mn*_y_* O_2_ (NCM)‐based cathodes stand out due to their competitive cost, compositional versatility, high capacity and energy density. Controlling the Ni/Co/Mn ratio is a key strategy for optimizing the balance between capacity, structural stability, and electrochemical performance. In this work, metal glycerolates synthesized via a solvothermal route were employed as hybrid organic–inorganic precursors to prepare NCM materials. The compositional variation, spanning from low‐ to high‐Ni content, enabled a direct correlation between Ni content, structural disorder, and electrochemical performance. Increasing the Ni content promoted Li^+^/Ni^2+^ cation mixing and structural disorder, resulting in reduced discharge capacity of Ni‐rich NCM cathodes. In contrast, low‐Ni NCM compositions exhibited improved structural ordering and electrochemical behavior, with Mg^2+^ doping effectively improving the stabilization of the LiNi_0.33_Co_0.33_Mn_0.33_O_2_ (NCM 333) structure and enhancing its rate capability and cycling stability. These findings provide important insights into the relationship between composition, structural ordering, and electrochemical behavior, while highlighting both the potential and current limitations of metal glycerolate‐based synthesis for NCM cathodes. Thus, metal glycerolates offer a basis for the rational design of cost‐effective and more sustainable energy storage materials.

## Graphical abstract

Metal glycerolates emerge as promising hybrid organic–inorganic precursors for NCM cathodes, revealing composition‐dependent structure–property relationships and offering a basis for the rational development of cost‐effective and more sustainable energy storage materials.

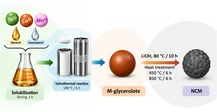

## 1 Introduction

The development of lithium‐ion batteries (LIBs) dates back to the 1970s and 1980s, gaining widespread attention after their commercial launch by Sony Corporation in the early 1990s [1, 2, 3]. Since then, they have become the dominant power source for portable electronic devices, electric vehicles (EVs), and stationary energy storage systems. This technological and scientific progress was further recognized in 2019, with the Nobel Prize in Chemistry awarded to John B. Goodenough, M. Stanley Whittingham, and Akira Yoshino for their pioneering contributions to the development of LIBs, a recognition that clearly led to greater investment and consequent expansion of research in the battery field. In addition, aligned with global efforts toward a sustainable and low‐carbon future, LIBs have become a focal point in the energy transition, playing a crucial role in advancing electric mobility (reducing transportation sector carbon emissions). Furthermore, they enable efficient renewable energy storage and support intelligent management of energy distribution and consumption at both small and large scales, thereby facilitating the integration of renewable energy sources such as wind and solar power into the electricity grid [4, 5, 6, 7, 8].

The success of LIBs as leading electrochemical energy‐storage systems is largely attributed to a combination of intrinsic advantages, including relatively high energy density, long cycle life, rechargeability, and lower environmental impact on a life‐cycle basis [9, 10, 11, 12, 13, 14, 15]. Despite these advantages, the recent advancement of electromobility, regarded as a central pillar of global decarbonization strategies, has intensified the demand for continued research aimed at developing next‐generation LIBs. This research focuses on improving electrochemical performance, enhancing stability (operational safety), and reducing manufacturing costs through the incremental optimization of established constituents and the exploration of novel materials, whether electrodes, electrolytes, or other key components [3, 16, 17, 18, 19, 20].

Among the several components of LIBs (cathode, anode, separator, electrolyte, and current collector), the cathode material has a significant impact on the electrochemical performance, safety, and cost [20, 21, 22, 23]. Therefore, considerable efforts have been devoted to developing and optimizing cathode materials. Indeed, advances in their design and synthesis, achieved through particle engineering and the tuning of surface chemistry, crystal structure, and morphology, have driven the evolution of both conventional and next‐generation systems. Moreover, the versatility and compositional diversity of these materials, along with the wide range of synthesis methods, such as solid‐state, coprecipitation, sol–gel, hydrothermal, and solvothermal processes, have enabled significant progress in the development of advanced electrode materials [19, 24, 25, 26, 27, 28, 29].

Herein, we demonstrate that metal glycerolates can serve as tailored hybrid organic–inorganic precursors for the synthesis of layered LiNi_1−*x*−*y*_Co_*x*_Mn_*y*_O_2_ cathodes (NCM). Metal glycerolates are formed through the deprotonation of glycerol hydroxyl groups, leading to alkoxide species with strong coordination ability toward metal centers [30]. These features make them suitable precursors for producing well‐structured hybrid materials that, upon calcination, decompose and oxidize to yield homogeneous and highly crystalline layered oxide phases, such as NCMs. NCM materials have emerged as mainstream cathode materials for modern LIBs, particularly for EVs, due to their competitive cost, compositional versatility, and potential for high capacity and energy density [26, 31, 32]. Their electrochemical performance arises from the synergistic effect of their constituent metals: Ni provides high specific capacity through reversible Ni^2+^/Ni^3+^/Ni^4+^ redox reactions; Co enhances electronic conductivity and structural integrity; and Mn, which remains mostly electrochemically inactive as Mn^4+^, contributes to thermal and structural stability [20, 33, 34, 35, 36]. Therefore, the proposed synthetic route, combined with compositional control, offers a simple and versatile pathway for producing layered NCM cathode materials with well‐defined composition and microstructure, enabling direct correlations between structure, electrochemistry, and stability. By integrating glycerol as a renewable, biodegradable, and nontoxic cosolvent and ligand source, this approach contributes to the development of environmentally responsible synthetic routes for layered oxide cathodes. In addition, it opens new perspectives for the design of glycerolate‐based precursors, representing an innovative and promising strategy for the synthesis of advanced cathode materials.

## 2 Experimental Section

### 2.1 Materials Preparation

The NCM precursors, that is, the metal glycerolates (M‐glycerolates), were synthesized via a solvothermal method [37]. Stoichiometric amounts of nickel (Ni), cobalt (Co), and manganese (Mn) nitrate salts (Sigma Aldrich) were weighed and added into an Erlenmeyer flask, in which defined amounts of isopropanol (Synth) and glycerol (Synth) were added. The molar ratio of metal salts to glycerol was 1:44, with isopropanol added at an approximate 1:5 (v/v) ratio relative to glycerol. The resulting metal salt suspensions were stirred for 1 h at room temperature, yielding a homogeneous solution. The solutions were then transferred into a Teflon‐lined stainless‐steel autoclave and heated at 180 °C for 6 h. The solvothermal reaction products, containing a precipitate and a supernatant, were collected, centrifuged, and the solids were washed with ethanol and dried at 60 °C for 6 h. The as‐prepared M‐glycerolate precursor materials were mixed with aliquots of LiOH⋅H_2_O (Sigma Aldrich) solution and dried at 80 ºC for c.a. 10 h and then subjected to a two‐step high‐temperature treatment in a controlled oxidizing atmosphere: a presintering step at 450 ºC for 6 h, followed by sintering at 850 °C for 6 h. Both steps were carried out at a heating rate of 5 °C/min and under synthetic airflow. Scheme 1 outlines the synthetic routes for the preparation of the precursor and NCM materials.

**SCHEME 1:**
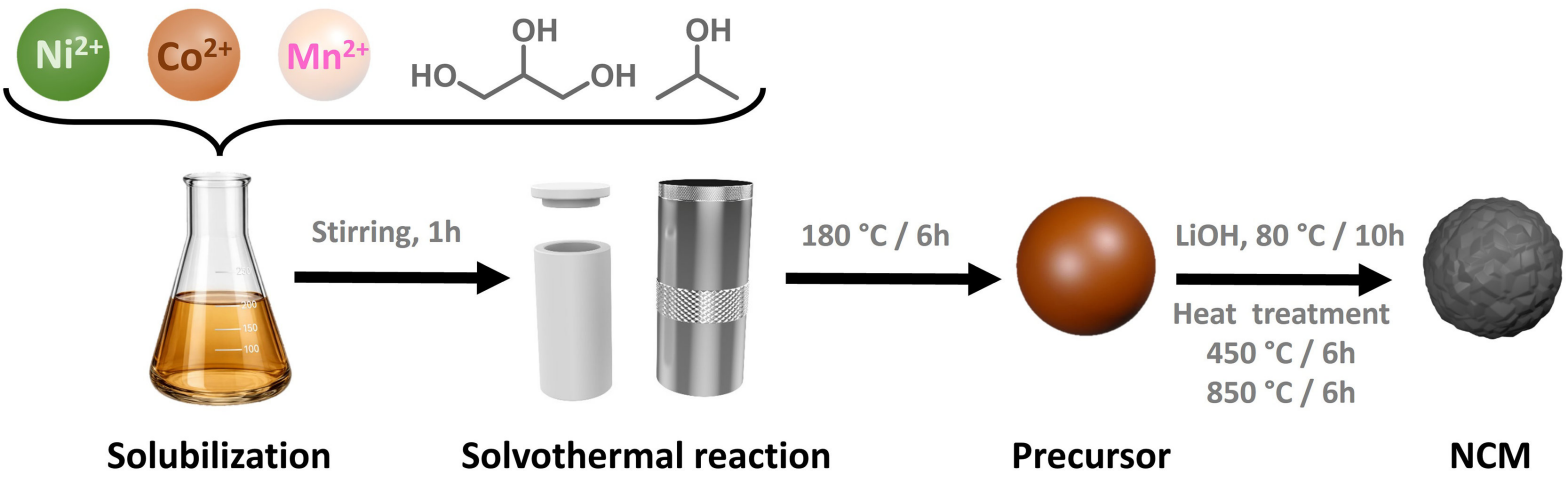
Illustration of the synthetic routes for the preparation of the M‐glycerolate precursor and NCM materials.

Magnesium (Mg) doping was carried out during the solvothermal synthesis; therefore, the obtained metal glycerolate precursor was already doped with Mg. For this purpose, 3 mol% of Mg was added to the initial metal solution, while 3 mol% of Co was correspondingly reduced to maintain the overall metal stoichiometry.

### 2.2 X‐Ray Diffraction

Powder X‐ray diffractograms of the LNO and NCM material samples were determined with a Bruker D8 Advance diffractometer using Cu–Kα radiation (1.5418 Å, 30 kV, 15 mA) employing a scan step of 0.05°. The Mg‐doped sample was measured using a different X‐ray diffraction (XRD) instrument, a Bruker D2 phase diffractometer using Cu–Kα radiation (1.5418 Å, 30 kV, ‍15 mA) employing a scan step of 0.05°. The lattice parameters of the samples were refined by the Rietveld method with General Structure Analysis Software (GSAS‐II).

### 2.3 X‐Ray Photoelectron Spectroscopy

X‐ray photoelectron spectroscopy (XPS) measurements were performed using a K‐Alpha XPS system (Thermo Fisher Scientific). The excitation energy was 1486.6 eV using an Al Kα X‐ray source, with a step of 0.1 eV, an acquisition time of 0.05 s, and *E*_pass_of 50 eV. The High‐resolution spectra were analyzed using CasaXPS 2.1 software. They were corrected using the carbon adventitious signal at 284.8 eV. To fit the 2p spectra of the metals, variations of the LA function were applied, with constraints on the FWHM and area ratio under a Shirley‐type baseline.

### 2.4 Scanning Electron Microscopy

Scanning electron microscopy (SEM) images were obtained with a JEOL JSM 7800F (FEG), coupled with energy‐dispersive X‐ray spectroscopy (EDS).

### 2.5 Electrochemical Measurements

In order to carry out electrochemical measurements, all the NCM samples were fabricated into cathodes for subsequent production of 2032‐type coin cells. Thus, NCM (as active material) was dispersed in N‐methyl‐2‐pyrrolidone (Sigma–Aldrich, 99.5%) with a polyvinylidene fluoride (Kynar HSV1800) binder, and then a conductive carbon black was added. The weight compositions of active material, binder, and conductive carbon were 8:1:1, respectively. The slurry was stirred and subsequently coated on an Al foil using a doctor blade. The coated electrodes were initially dried at 90 °C for 3 h in the doctor‐blade coating system, followed by further drying in a vacuum oven at 80 °C for 10 h to ensure complete solvent removal.

The dried NCM cathodes were prepared and punched with a 16 mm diameter disc and then assembled into 2032‐type coin cells in an argon‐filled glovebox. Lithium metal was used as the counter electrode, and polypropylene (PP) membrane served as the separator. The electrolyte consisted of 1 M LiPF_6_ (Sigma–Aldrich) dissolved in a 1:1 mixture of ethylene carbonate (EC) and ethyl methyl carbonate (EMC). The electrochemical performance was evaluated in the potential range of 2.7–4.3 V versus Li/Li^+^. Galvanostatic charge–discharge activation was performed at 0.1 C (1C = 170 mA g^−1^) for three cycles. Rate performance was then evaluated from 0.1 to 5.0 C, with currents based on the capacity measured during activation (129, 113, 94, 65, and 60 mA g^−1^ for samples NCM 333, NCM 244, NCM 631, NCM 622, and NCM 613, respectively), within a voltage range of 2.7–4.3 V at room temperature. Cycling stability was performed using the capacity of 129 mAh g^−1^ at 1 C for 100 cycles, after a 0.1 C activation cycle, under the same voltage window.

Cyclic voltammetry (CV) was performed between 2.7 and 4.3 V versus Li/Li^+^ at scan rates of 0.1, 0.2, 0.3, 0.4, 0.5, and 1.0 mV s^−1^. Electrochemical impedance spectroscopy (EIS) was conducted using a frequency range of 10 kHz to 100 mHz and a small‐amplitude sinusoidal perturbation of 10 mV. All the electrochemical measurements were conducted using a multichannel Neware Battery Tester System (CT‐ZWJ‐4’S‐T‐1U) and a Bio‐Logic Battery Cycling and Testing system (BCS‐810 and BCS‐905).

## 3 Results and Discussion

### 3.1 Preparation of the NCM Samples

The solvothermal synthetic route simplifies precursor preparation and offers better control over morphology, reduced particle size, and lower impurity levels, while the two‐step high‐temperature treatment promotes structural ordering, which tends to improve electrochemical performance. This combination reduces the dependence on the strict precipitation control required in coprecipitation methods and contributes to controlled particle size and enhanced dispersion of the components, offering advantages over the sol–gel approach in terms of process simplicity and precursor handling.

A comparison with previously reported glycerol‐assisted synthesis routes highlights important differences in the role played by glycerol and in the nature of the precursor obtained. Glycerol‐assisted routes have previously been explored for the synthesis of layered cathodes, with glycerol acting as a solvent, complexing agent, morphology‐directing agent, or fuel [38, 39, 40, 41]. Ding et al. [38] and Fang et al. [39] employed glycerol‐assisted solvothermal synthesis to regulate the morphology and crystal growth of NCM materials. In these approaches, glycerol was used as a complexing agent, together with urea as a precipitating agent, leading to the formation of carbonate‐based precursors. Although these studies demonstrate the versatility of glycerol in the synthesis of NCM materials, the role of glycerol in such approaches differs from that investigated in the present work. In addition, the carbonate precursors reported in these studies differ from the M‐glycerolate precursors investigated here in terms of their chemical nature, morphology, and structural characteristics.

The M‐glycerolate precursors were successfully synthesized via the solvothermal method. The amount of glycerol used was consistent with values previously reported for metal glycerolate synthesis [42, 43]. Therefore, the ethanol washing should effectively remove any unreacted or free glycerol. From the two‐step high‐temperature treatment, seven metal‐layered oxide Li(TM)O_2_ were synthesized: LiNiO_2_ (LNO, 100% Ni), LiNi_0.8_Co_0.1_Mn_0.1_O_2_ (NCM 811), LiNi_0.6_Co_0.2_Mn_0.2_O_2_ (NCM 622), LiNi_0.6_Co_0.3_Mn_0.1_O_2_ (NCM 631), LiNi_0.6_Co_0.1_Mn_0.3_O_2_ (NCM 613), LiNi_0.33_Co_0.33_Mn_0.33_O_2_ (NCM 333), and LiNi_0.2_Co_0.4_Mn_0.4_O_2_ (NCM 244). This compositional variation was designed to systematically investigate the effect of Ni content, accompanied by inherent changes in Co and Mn proportions, on the morphological, structural, and electrochemical properties of NCM materials.

### 3.2 Morphological and Elemental Characterization

The morphology of conventional M‐glycerolates consisted of spherical particles (Figure 1a), which can also be found agglomerated depending on the metal composition and proportion, as shown in Figure S1 of the Supporting Information. After a two‐step high‐temperature treatment, SEM analysis revealed that the LNO particles have a nonuniform morphology, with a brittle‐like appearance (Figure 1b). On the other hand, the NCM samples consisted of micrometer‐sized polycrystalline secondary particles with spherical or near‐spherical morphology, formed by the aggregation of polyhedral primary particles (Figure 1c–f). This type of morphology is very common in NCM materials and can provide advantages, such as facilitating Li^+^ transport and enhancing specific capacity. In addition, the secondary particles of NCM 333 tend to exhibit a few cavities, which can enhance Li^+^ transport. However, these cavities may also increase surface area and interfacial reactivity, potentially reducing the first‐cycle coulombic efficiency and volumetric energy density [44, 45]. The EDS elemental mapping of the three NCM samples revealed that Ni, Co, Mn, and O are homogeneously distributed throughout the particles, indicating uniform compositional distribution, as shown in Figure 2.

**FIGURE 1:**
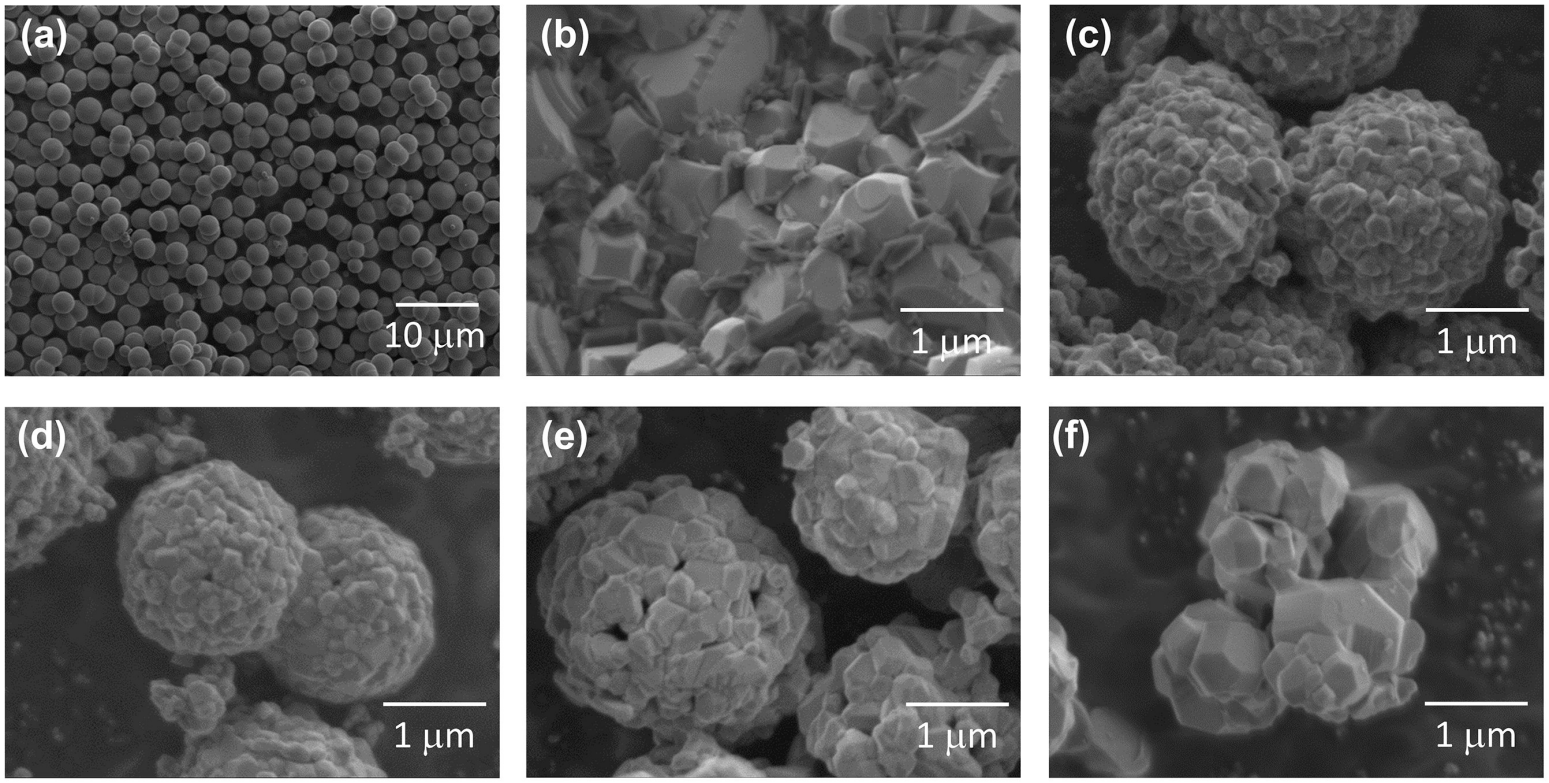
SEM images of (a) M‐glycerolate (Ni:Co:Mn = 1:1:1), (b) LNO, (c) NCM 811, (d) NCM 622, (e) NCM 333, and (f) NCM 244.

**FIGURE 2:**
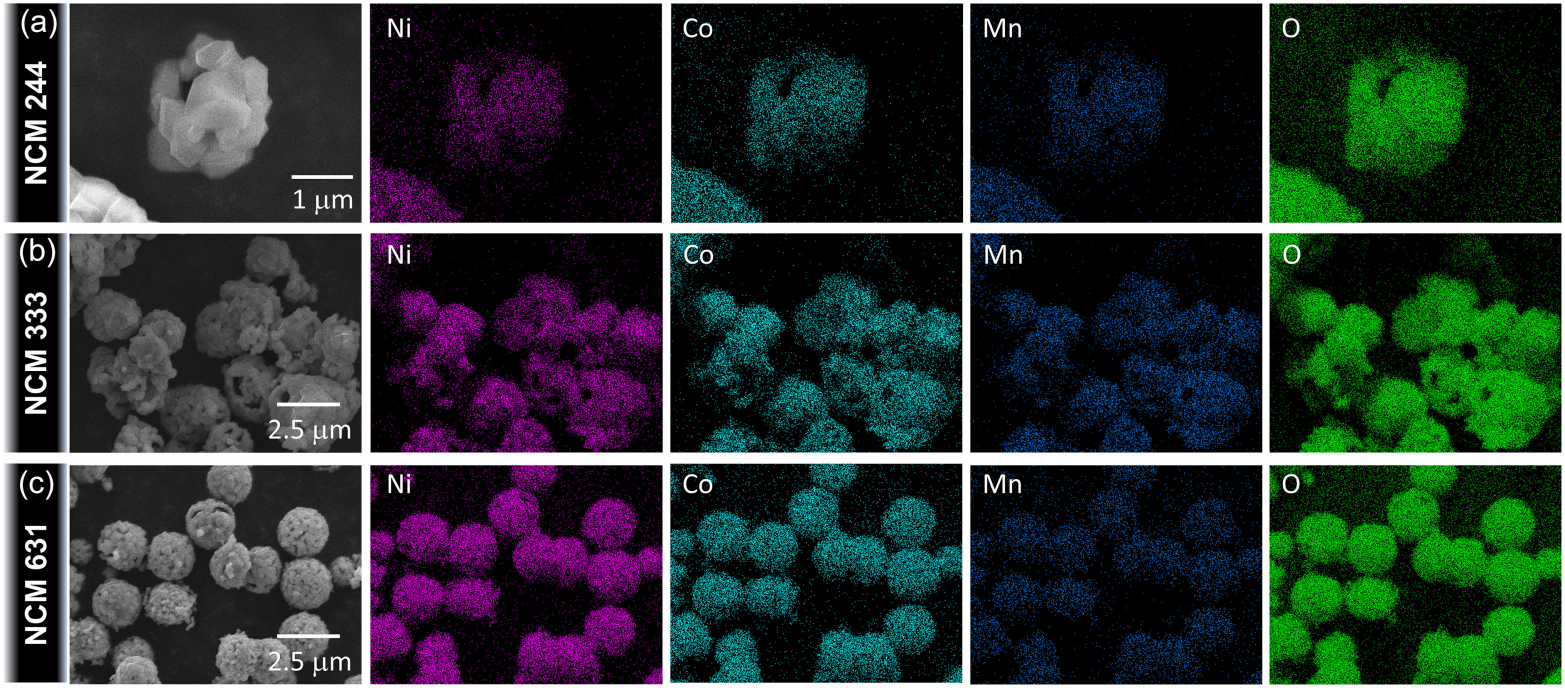
(a) SEM‐EDS mapping of (a) NCM 244, (b) NCM 333, and (c) NCM 631.

### 3.3 Structural Characterization

XRD patterns of the synthesized samples are shown in Figure 3a and Figure S2. Both LNO and NCM samples crystallize in a layered α‐NaFeO_2_‐type structure, belonging to the rhombohedral crystal system (space group (166) *R‐3m*), consistent with the reference patterns (PDF# 01‐084‐4264) and (PDF# 01‐074‐4671), respectively [46, 47]. The diffraction patterns of NCM samples with low Ni content showed well‐defined peaks, including split twin peaks (006/102) and (018/110), as shown in Figure 3b, indicating good crystallinity and a highly ordered hexagonal layered structure. In contrast, increasing Ni content led to reduced peak splitting, suggesting a lower degree of layered ordering. Notably, most of the diffraction peaks shift slightly toward higher angles with decreasing Ni content, indicating lattice contraction in the a–b plane due to the smaller ionic radii of Mn^4+^ (0.53 Å) and Co^3+^ (0.54 Å) compared with Ni^2+^ (0.69 Å). On the other hand, the (003) peak position exhibits a nonmonotonic shift, reflecting variations in the c‐axis parameter, which are influenced by the interplay between cation mixing (Li^+^/Ni^2+^) and changes in layered ordering [48, 49].

**FIGURE 3:**
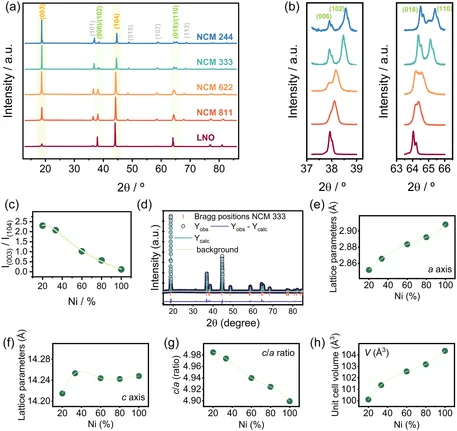
(a) XRD patterns of LNO and NCM samples; (b) zoom‐in of the (006/102) and (018/110) peak regions; (c) relationship between the I_(003)_/I_(104)_ intensity ratio and Ni content in the NCM; (d) example of Rietveld refinements of NCM 333; Refined structural parameters of the sample series with (e) *a* and (f) *c* lattice parameters, (g) *c*/*a* ratio, and (h) unit cell volume, all versus the Ni content in the NCM.

The intensity ratio of the (003) to (104) peaks (I_(003)_/I_(104)_) serves as a crucial indicator of cation mixing between Li^+^ and Ni^2+^; a higher ratio reflects a lower degree of cation mixing [50, 51]. As shown in Figure 3c, there was a significant mixing of Li^+^ and Ni^2+^ in the NCM samples with Ni content from 60%, particularly more pronounced in the LNO sample, where the intensity of the (003) reflection nearly disappeared. As reported by Kalyani and Kalaiselvi [52], synthesizing stoichiometric LiNiO_2_ is quite challenging, so it is more accurately represented as a solid solution denoted by Li_*x*_Ni_2‐x_O_2_ (0 ≤ *x* ≤ 1), with LiNiO_2_ (*x* = 1) and NiO (*x* = 0) as the end members. As x decreases, the concentration of Ni^2+^ ions in the lithium layers increases, which in turn reduces the availability of effective lithium ions in the structure. This reduction leads to greater polarization in the cell, thereby impacting on the electrochemical capacity and rate capability of the cathode. In addition, the lithium loss during high‐temperature calcination, due to its high vapor pressure, leads to nonstoichiometric samples and cation mixing (Ni occupying Li sites), which lowers the initial capacity and accelerates capacity fade during cycling [52]. The presence of Ni^2+^ in the lithium layers also hinders Li^+^ diffusion, reduces electrical conductivity, and induces structural disorder while promoting irreversible phase transitions [52, 53]. Consequently, the electrochemical performance of LiNiO_2_ is highly sensitive to synthesis conditions, making strict control of atmosphere and temperature essential to minimize these adverse effects. Indeed, for the material with 100% Ni content synthesized here, the XRD result indicated the formation of an electrochemically inactive phase, possibly Li_2_Ni_8_O_10_, which is known to result from complete cation mixing, leading to the loss of the layered structure necessary for lithium intercalation and thus rendering the material unsuitable as a cathode material.

To quantitatively evaluate the structural evolution of layered oxide materials in the diffraction patterns, Rietveld refinement was performed on the XRD data of NCM samples using a structure model with the space group *R‐3m*, indexed as a hexagonal lattice. In this arrangement, Li ions occupy the 3a site, the transition metal ions (M = Mn, Co, and Ni) are located at the 3b site, and the O ions occupy the 6c site [54, 55, 56]. A representative example of the refinement quality, showing the agreement between the observed and calculated intensities along with the difference curve, is presented in Figure 3d for NCM 333. The refined structural parameters for the complete series are summarized in Table 1, and their dependence on Ni content is illustrated in Figure 3e–h. As the Ni content decreases from 100% (LNO) to 20% (NCM 244), a systematic contraction of the lattice parameters is observed. The *a* parameter decreases from 2.90822(9) Å in LNO to 2.85170(11) Å in NCM 244, while the c parameter does not exhibit a linear trend; it initially increases from 14.2148(7) Å in NCM 244–14.2533(4) Å in NCM 333, then decreases to approximately 14.24 Å for higher Ni contents (NCM 622, NCM 811, and LNO). This behavior suggests that while low‐Ni compositions (NCM 244 and NCM 333) benefit from enhanced layered ordering and expanded interlayer spacing, further increases in Ni content led to structural perturbations, including increased Li^+^/Ni^2+^ cation mixing, which partially collapses the interlayer distance. Accordingly, the unit cell volume contracts from 104.3617(14) Å^3^ in LNO to 100.110(8) Å^3^ in NCM 244. This trend is attributed to the progressive substitution of larger Ni^2+^ ions (0.69 Å) by smaller Mn^4+^ (0.53 Å) and Co^3+^ (0.54 Å) ions, which promotes lattice densification in the *ab*‐plane. Furthermore, the *c*/*a* ratio increases with decreasing Ni content, from 4.90 in LNO to 4.98 in NCM 244, indicating a higher degree of layered ordering and reduced Li^+^/Ni^2+^ cation mixing. This is consistent with the observed increase in the I_(003)_/I_(104)_ intensity ratio, which reaches 2.08 for NCM 333 and 2.30 for NCM 244, confirming improved structural integrity and lower disorder in low‐Ni compositions. The Ni occupancy in the Li layer (3a site) increases progressively with increasing Ni content, from 0.027 for NCM 244–0.310 for NCM 811, indicating a correlation between Ni content and cation mixing. Concurrently, the Ni occupancy at the 3b site increases from 0.173 (NCM 244) to 0.783 (NCM 811), consistent with the expected redistribution of Ni between the two sites. For the LNO sample, the Ni^2+^ occupancy at the 3a site is 0.256, slightly lower than that of NCM 811, while its occupancy at the 3b site reaches 0.820.

**TABLE 1:** Refined structural parameters of the unit cell for the sample series.

Samples	Ni, %	*a* parameter, Å	*c* parameter, Å	Volume, Å^3^	*c*/*a* ratio	R_wp_, %	*χ* ^2^	Ni Occup.	
								3a	3b
NCM 244	20.0	2.85170(11)	14.2148(7)	100.110(8)	4.98	9.75	10.79	0.027	0.173
NCM 333	33.3	2.86566(7)	14.2533(4)	101.367(5)	4.97	6.107	4.35	0.060	0.273
NCM 622	60.0	2.88352(4)	14.24387(17)	102.5665(17)	4.94	4.744	3.84	0.144	0.541
NCM 811	80.0	2.89227(9)	14.24256(28)	103.180(5)	4.92	4.675	4.21	0.310	0.783
LNO	100.0	2.90822(9)	14.2480(4)	104.3617(14)	4.90	8.410	13.64	0.256	0.820

### 3.4 Electrochemical Performance

Since the LNO structure is not favorable as an active material and considering the interest in ternary NCM‐type cathodes, the activation processes at 0.1 C were carried out only for the NCM samples. As shown in Figure 4a, it is evident that the initial discharge capacity obtained for the NCM 811 sample (<10 mAh g^−1^) is quite poor for an active material. This result is consistent with the structural analysis, confirming that the elevated cation mixing in the NCM samples with higher Ni content contributes to their low capacity. Indeed, the NCM 333 and NCM 244 cathodes delivered the best initial discharge capacities among all compositions (Figure 4a), 129 and 113 mAh g^−1^, respectively. Although NCM 631 exhibited a relatively high initial capacity of 94 mAh g^−1^ (Figure 4a), its rate performance at different current densities (0.1, 0.2, 0.5, 1.0, 2.0, and 5.0 C) decreased drastically (Figure 4b). On the other hand, the NCM 333 and NCM 244 cathodes displayed good rate capability across current densities ranging from 0.1 to 5.0 C (Figure 4b). They demonstrated the highest and most stable discharge capacities, along with high initial coulombic efficiency (88.2% for NCM 333 and 93.9% for NCM 244) and good capacity retention (43% and 60% at 5 C for NCM 333 and NCM 244, respectively). All these findings are in agreement with the structural information discussed above. Therefore, such electrochemical performance originates from the favorable crystal architecture, which enhances ionic mobility and structural stability by providing more active sites and effectively buffering the strain generated during lithiation/delithiation.

**FIGURE 4:**
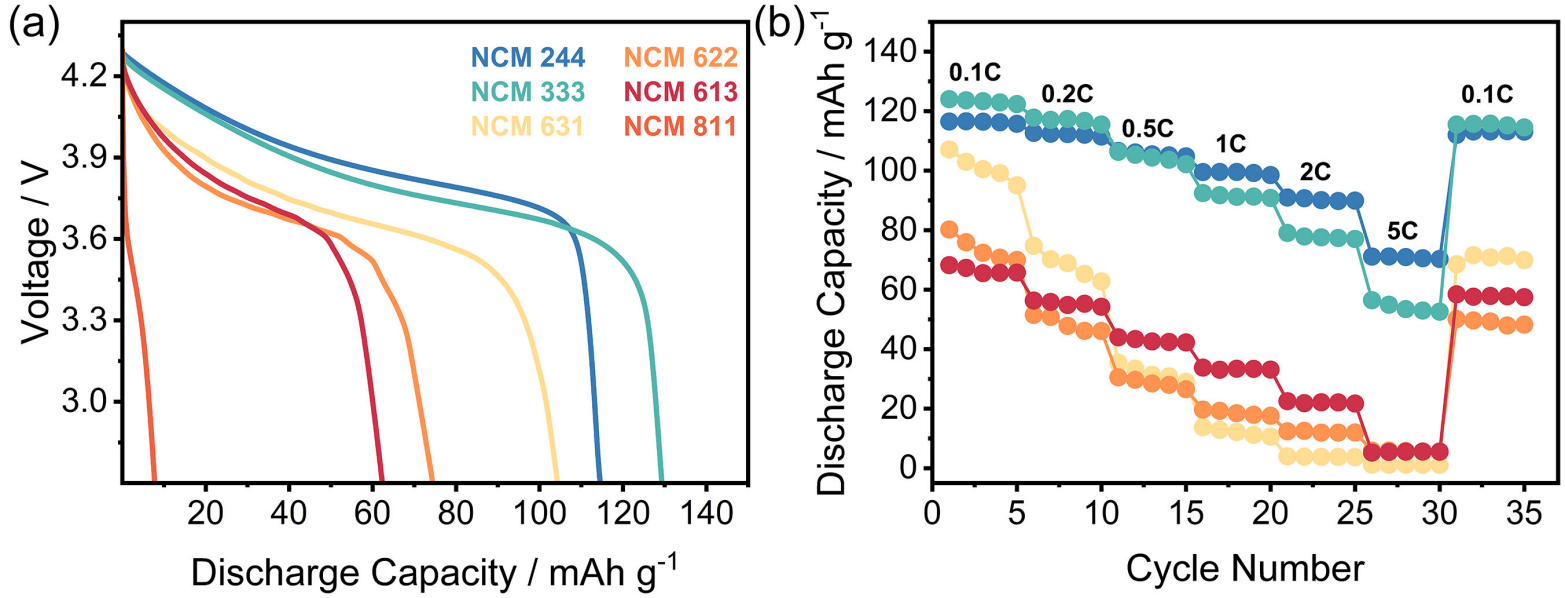
(a) Initial discharge curves of the NCM samples measured at 0.1 C and (b) rate performance measured at different C‐rates.

The specific capacity of layered metal oxide cathodes generally increases with increasing Ni content as the Ni^3+^/Ni^4+^ redox couple contributes significantly to the charge–discharge process. This, in principle, allows higher lithium‐ion utilization and improved electrochemical performance [26, 57]. However, for the samples obtained in this work, the opposite behavior was observed: the high‐Ni NCM cathodes exhibited poorer capacity and rate performance. This deviation can be attributed primarily to the increased presence of Ni^2+^ ions at Li sites (cation mixing), as evidenced by the structural analysis. This effect may also be accompanied by additional structural disorder, which can influence the lattice parameters. These features are known to hinder Li^+^ diffusion and may compromise the structural integrity of the cathode. In addition, although the morphologies of the NCM samples are very similar, minimizing the comparative influence of morphology on electrochemical performance, the presence of a few cavities in the NCM 333 particles may enhance Li‐ion transport and account for the superior rate performance observed.

It is important to emphasize that the same general thermal treatment was applied to the different NCM compositions in this study to enable a consistent comparison of the effect of Ni content on their structural and electrochemical properties. The poorer electrochemical performance observed for the Ni‐rich compositions, particularly the very limited capacity of NCM 811, is discussed primarily in relation to the increased Li^+^/Ni^2+^ cation mixing and structural disorder observed with increasing Ni content. However, since the calcination conditions were not independently optimized for each composition, the possible contribution of nonoptimal thermal treatment to the electrochemical performance of the Ni‐rich samples cannot be excluded. Therefore, the present results should not be interpreted as evidence of an inherent limitation of the M‐glycerolate route for Ni‐rich NCM materials.

### 3.5 Mg‐Doping Studies and Electrochemical Comparison

Considering the properties of NCM 333, that is, lower cation mixing, higher specific capacity, good rate performance, and higher Ni content and consequently lower Co content than NCM 244, the NCM 333 cathode was chosen for further studies. Therefore, its stability and electrochemical properties were further evaluated with the aim of improving them through metal doping with magnesium ions, Mg^2+^. The incorporation of multivalent metal cations into NCM cathodes can affect the structural and surface characteristics of the material. By decreasing Li^+^/Ni^2+^ disorder and facilitating Li^+^ mobility, metal doping enhances bulk stability while also alleviating stress‐induced cracking. Furthermore, segregation phenomena during calcination can alter the surface chemistry, lower reactivity, and provide additional surface protection [58]. Therefore, during the solvothermal synthesis process, 3% of Mg nitrate salt was added together with the other metal salts (33% of Ni, 33% of Mn, and 30% of Co) in order to obtain the Mg‐doped NCM 333 sample. The morphology of NCM 333 remains unchanged with Mg^2+^ doping (Figure 5a), following the formation of secondary particles from primary particles. Besides, EDS mapping confirmed the homogeneous distribution of each metallic element, also showing that the doping was successful (Figure 5b).

**FIGURE 5:**
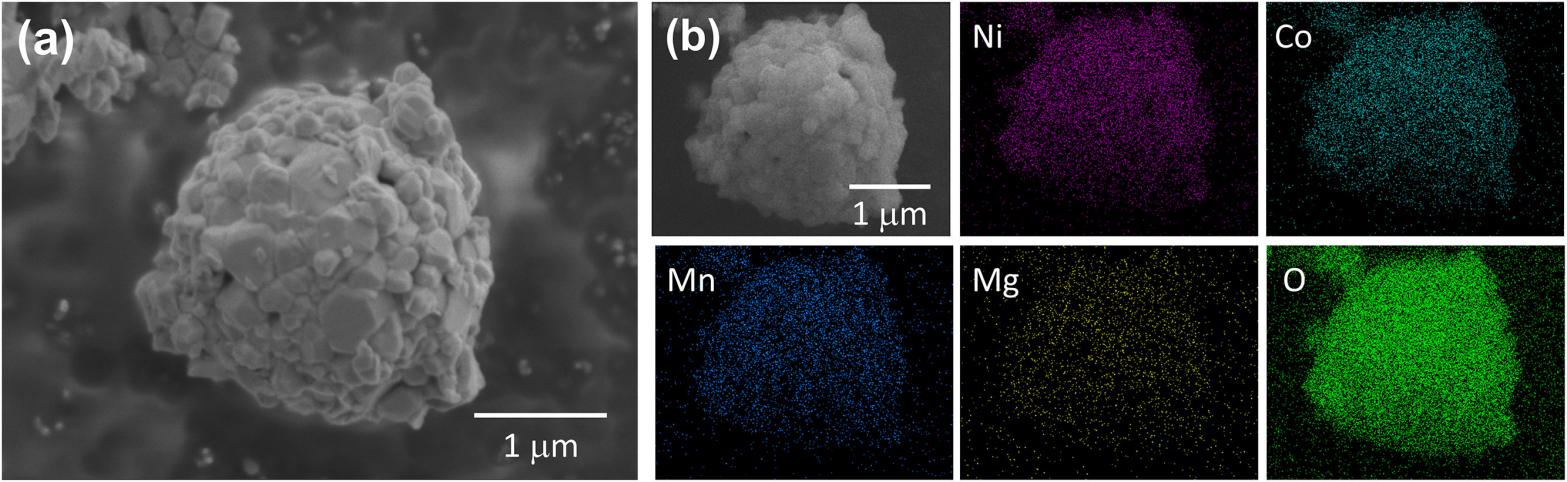
(a) SEM image and (b) SEM‐EDS mapping of Mg‐NCM 333.

XPS analysis was performed to investigate the surface chemical composition of NCM 333 and Mg‐doped NCM 333. Figures 6a and S3 present the survey spectra for Mg‐doped NCM 333 and NCM 333, respectively, and Figure 6b–e presents the corresponding high‐resolution spectra. Photoelectron peaks corresponding to Ni (2p_3/2_), Mn, Co, Mg, O, and C elements were identified in the survey spectrum. The analysis of NCM 333 showed no significant shifts in the Ni, Co, and Mn 2p core‐level spectra compared to the Mg‐doped NCM 333 sample, indicating that Mg incorporation does not substantially alter the oxidation states of the NCM transition metals.

**FIGURE 6:**
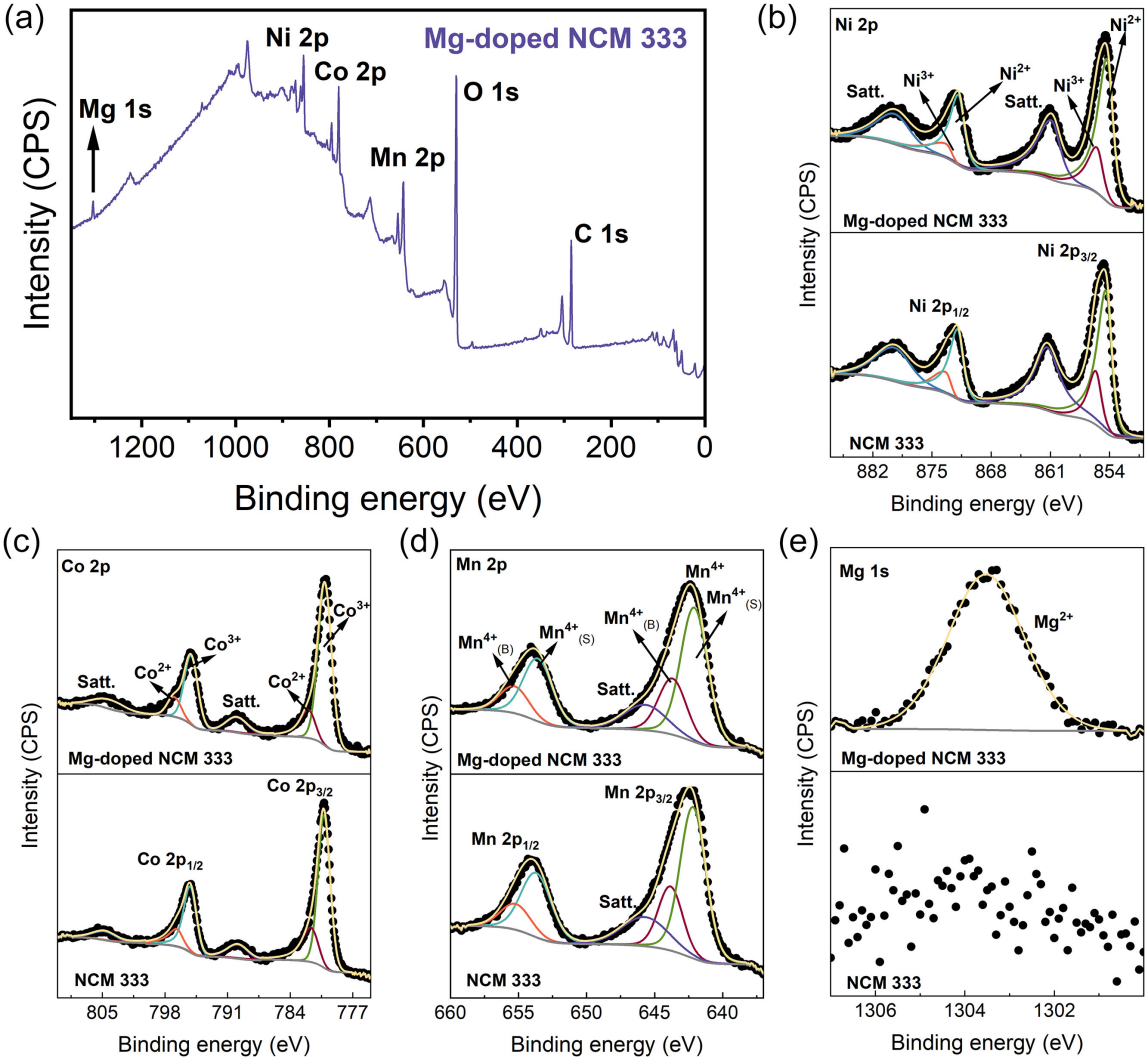
(a) Survey XPS spectrum of the Mg‐doped NCM 333; (b) Ni 2p, (c) Co 2p, (d) Mn 2p, and (e) Mg 1s XPS spectra for NCM 333 and Mg‐doped NCM 333.

The high‐resolution spectrum of Ni 2p (Figure 6b) presented fitted peaks around 854.3 (Ni 2p_3/2_) and 871.9 eV (Ni 2p_1/2_) assigned to the Ni^2+^ state, while the peaks located at 855.6 eV (Ni 2p_3/2_) and 873.3 eV (Ni 2p_1/2_) correspond to Ni^3+^ [59]. Characteristic satellite peaks were observed at 860.9 and 879.4. The Co 2p spectra (Figure 6c) were fitted with four components. The Co^3+^ species was identified at 780.1 eV (Co 2p_3/2_) and 795.1 eV (Co 2p_1/2_), while the Co^2+^ was associated with the peaks at 781.7 eV (Co 2p_3/2_) and 796.9 eV (Co 2p_1/2_). Two satellite peaks were also observed at 790.0 and 804.2 eV. The Mn 2p high‐resolution spectra for the materials (Figure 6d) exhibited a Mn 2p_3/2_ peak at 642.4 eV, which is associated with the Mn^4+^ state (MnO_2_) [60, 61]. The spin–orbit splitting of 11.7 eV between the 2p_3/2_ and 2p_1/2_ peaks also confirms that Mn^4+^ is the predominant oxidation state [60]. Thus, the Mn 2p spectra were fitted with four components corresponding to the MnO_6_ octahedral structure of Mn^4+^. The binding energies of 642.1 eV (Mn^4+^_(S)_ 2p_3/2_) and 653.8 eV (Mn^4+^_(S)_ 2p_1/2_) are correlated to the loose surface structure around the MnO_6_ octahedra, which is caused by the presence of oxygen vacancies (O_v_), resulting in weaker Mn─O bonds. On the other hand, the binding energies of 643.8 eV (Mn^4+^_(B)_ 2p_3/2_) and 655.7 eV (Mn^4+^_(B)_ 2p_1/2_) are assigned to bulk MnO_6_ octahedra, where the Mn─O bonds are stronger than in regions containing structural defects [62]. The Mg 1s high‐resolution spectra (Figure 6e) confirmed the presence of Mg^2+^ in the Mg‐doped NCM 333 sample. In addition, the insertion of Mg^2+^ into the NCM structure did not lead to any change in the Ni^3+^/Ni^2+^ ratio relative to NCM 333 as both samples exhibited a ratio of 0.32. This suggests that the degree of Li^+^/Ni^2+^ cation mixing remained comparable to that of NCM 333, indicating that Mg^2+^ insertion did not substantially modify the Ni oxidation state distribution at the material surface. The O 1s high‐resolution spectra (Figure S4) pointed to the presence of the M─O bond, located at 529.5 eV, and the oxygen vacancies (peak at 531.4 eV), while the band at 533.1 is assigned to adsorbed water on the material surface.

CV curves of NCM 333 and Mg‐doped NCM 333 at 0.1 mV s^−1^ in the voltage range of 2.7–4.3 V are shown in Figure 7a. Both samples exhibit a pair of broad anodic and cathodic peaks, arising mainly from the overlapping Ni^2+^/Ni^3+^, Ni^3+^/Ni^4+^ and Co^3+^/Co^4+^ redox reactions. The separation between the anodic and cathodic peak potentials (Δ*E*_p_) reflects the electrochemical polarization during lithiation and delithiation, which a smaller Δ*E*_p_ indicates lower polarization and faster reaction kinetics [50, 63, 64]. In this regard, the Mg‐doped NCM 333 shows a smaller peak separation (c.a. ‍0.14 V) compared to NCM 333 (Δ*E*_p_ ~ 0.16 V), suggesting improved electrochemical reversibility and facilitated charge–transfer dynamics.

**FIGURE 7:**
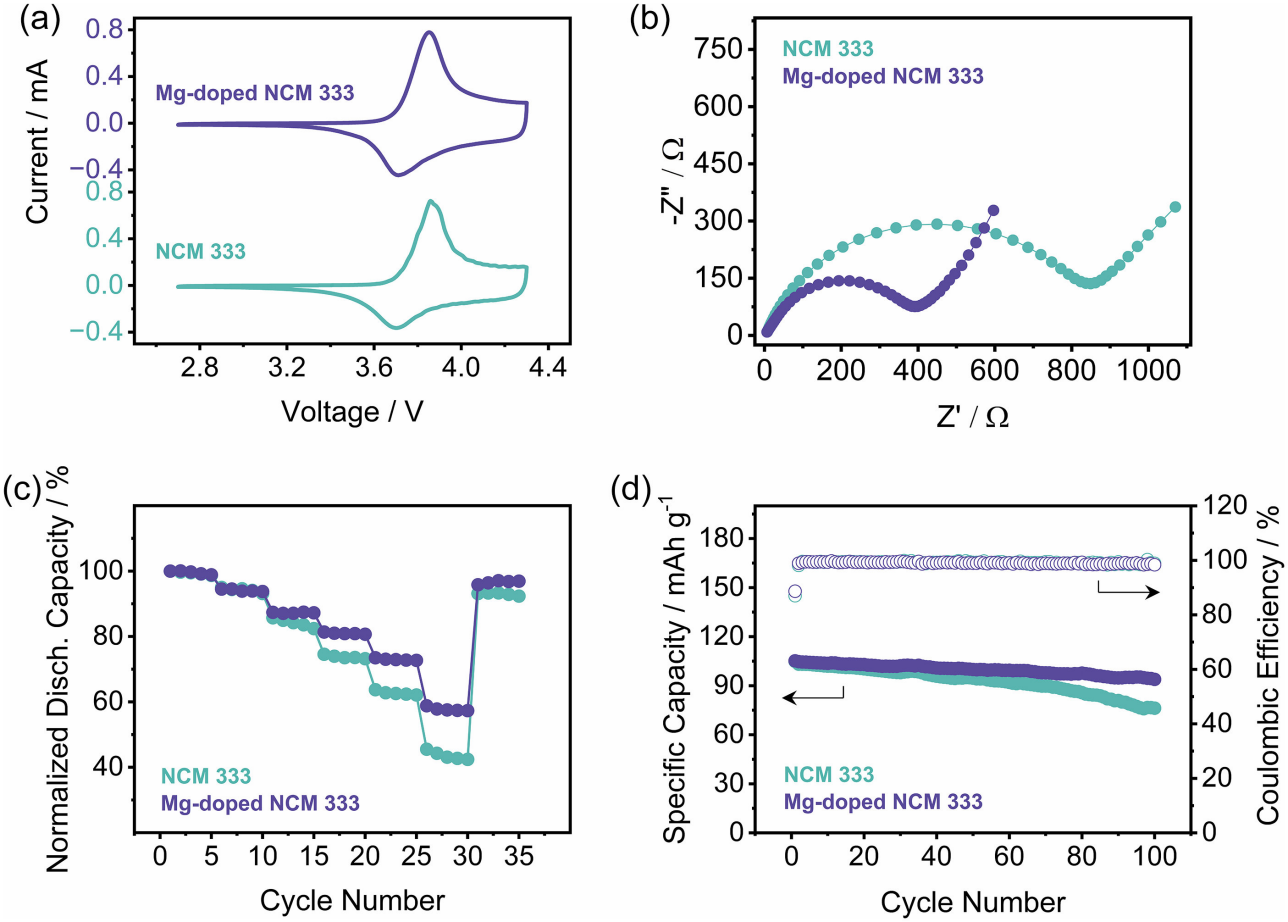
(a) Cyclic voltammetry of NCM 333 and Mg‐doped NCM 333; (b) Nyquist plots; (c) Normalized rate performance measured at different C‐rates; and (d) cycling performance at 1C.

Beyond the peak positions, the Mg‐doped sample also shows a larger voltammetric area (i.e., higher integrated current over the potential sweep), indicating a greater overall charge involved in the redox process under identical conditions. This behavior is consistent with a better Li^+^ (de)intercalation process, where a larger fraction of electrochemically active sites becomes more accessible. Mechanistically, Mg doping suggests enhanced transport and interfacial processes through multiple effects: (i) improved electronic/ionic percolation within the composite electrode and reduced local overpotentials; (ii) mitigation of Li^+^ diffusion limitations by lowering the effective diffusion resistance (e.g., via subtle structural stabilization, reduced cation mixing, and/or improved Li^+^ pathways in the layered lattice); and (iii) modification of the electrode/electrolyte interface, which can alter the balance between faradaic insertion and non‐faradaic contributions. In particular, Mg incorporation indicates an increase in the overall voltammetric response, changing how charge is stored and transferred during the potential sweep. The enhanced response may be associated with improved interfacial charge–transfer kinetics and/or a greater contribution of surface‐controlled electrochemical processes, facilitating Li^+^ intercalation/deintercalation.

To further support the interpretation of the enhanced voltammetric response, the cathodic branch of the CV curves was quantitatively integrated at different scan rates. The negative‐current region was considered because it is associated with the discharge process. As shown in Table S1, Mg‐doped NCM 333 exhibited higher cathodic discharge capacity than pristine NCM 333 over the entire scan‐rate range. At 0.1 mV s^−1^, the discharge capacity increased from 111.43 to 125.29 mAh g^−1^, while at 1.0 mV s^−1^ it increased from 79.06 to 96.85 mAh g^−1^. Considering all scan rates, the average CV‐derived discharge capacity increased from 97.96 to 113.50 mAh g^−1^, corresponding to an average improvement of approximately 15.9%. This increase in cathodic charge indicates that Mg incorporation enhances the electrochemically accessible discharge capacity and improves the utilization of redox‐active sites during Li^+^ intercalation/deintercalation. Moreover, the improvement observed at different scan rates, especially under faster polarization conditions, suggests that Mg doping mitigates kinetic limitations. Therefore, the enhanced CV response is more appropriately attributed to improved faradaic accessibility, lower polarization, and more favorable interfacial charge–transfer kinetics. Although the scan‐rate‐dependent behavior may indicate that both surface‐controlled/capacitive‐like processes and diffusion‐controlled faradaic processes are affected by Mg incorporation, the present data do not allow a quantitative separation of these contributions. Thus, no specific assignment to increased electrical double‐layer capacitance (EDLC) or quantified non‐faradaic charge storage is made.

To further investigate the origin of the enhanced CV response, the anodic and cathodic peak currents were extracted from the CV curves at different scan rates to provide a more direct comparison of the electrochemical response. As shown in Table S2, Mg‐doped NCM 333 exhibited higher anodic and cathodic peak currents over the entire scan rate range. The anodic peak current increased by approximately 3.9%–17.3%, whereas the absolute cathodic peak current increased by approximately 18.1%–39.7%, indicating improved charge/discharge kinetics under dynamic polarization conditions.

The scan rate dependence of the peak current was further evaluated using the relationship *i* = a*ν* ^*b*^. The b‐values obtained for pristine NCM 333 were 0.76 and 0.69 for the anodic and cathodic processes, respectively, while Mg‐doped NCM 333 exhibited b‐values of 0.80 and 0.76. These values suggest mixed kinetic behavior, involving both diffusion‐controlled faradaic Li^+^ intercalation/deintercalation and surface‐controlled/capacitive‐like contributions. However, since no quantitative capacitive/diffusion‐controlled current separation was performed, no specific assignment to increased EDLC or quantified non‐faradaic charge storage was made. Therefore, the enhanced CV response of Mg‐doped NCM 333 is more conservatively attributed to reduced polarization, higher electrochemically accessible charge, and improved apparent charge–transfer kinetics.

This was further supported by EIS, which revealed that the Mg‐doped NCM 333 sample exhibits a significantly smaller semicircle in the high‐ to medium‐frequency region, corresponding to reduced charge–transfer resistance (Rct), suggesting enhanced interfacial charge–transfer processes and improved ionic/electronic conductivity (Figure 7b).

The I_(003)_/I_(104)_ intensity ratio was found to be slightly lower for the Mg‐doped NCM 333 sample (I_(003)_/I_(104)_ = 2.02, from XRD pattern in Figure S5) than for NCM 333 (I_(003)_/I_(104)_ = 2.08), indicating only a very slightly higher degree of Ni ion occupancy in the lithium layer. This observation is consistent with the XPS results, which showed an unchanged Ni^3+^/Ni^2+^ ratio (0.32) for both samples, indicating that Mg doping did not significantly alter the Ni oxidation state. Thus, the Mg‐doped NCM 333 exhibited electrochemical performance comparable to that of NCM 333, with high discharge capacity (129 mAh g^−1^).

In addition, the Mg‐doped sample showed improved rate capability, with a gain of c.a. 15% in capacity retention at 5C (Figure 7C), and enhanced electrochemical stability, maintaining stable cycling performance for up to 100 cycles at 1C (Figure 7d). Indeed, after 100 cycles at 1C, both NCM 333 and Mg‐doped NCM 333 maintain high reversible capacities of 76.2 and 93.9 mAh g^−1^, respectively, with corresponding to capacity retentions of ~72% and 89%. This improved cycling stability is likely associated with the structural stabilization effects of Mg incorporation in layered cathode materials. Li et al. [48] attributed these effects to the pillar effect of Mg in the Li layers and its interactions with oxygen, which can mitigate structural degradation during cycling. In addition, Sattar et al. [65] reported a higher Mg─O bond energy than that of Ni─O, suggesting that Mg incorporation may contribute to reduced structural distortions in Ni‐rich NCM cathodes. Possibly, these mechanisms may contribute to the enhanced cycling stability observed in the present study. Indeed, as XRD data suggest, the relatively small variation in the I_(003)_/I_(104)_ ratio indicates that Mg doping did not substantially modify the degree of cation mixing in the layered structure. Therefore, in this work, the improved electrochemical stability observed for the Mg‐doped sample is unlikely to be primarily associated with a reduction in Li^+^/Ni^2+^ cation mixing. This improvement is consistent with the reduced polarization inferred from CV, in which the Mg‐doped NCM 333 exhibited a smaller Δ*E*_p_ and a larger voltammetric area, indicating a more efficient Li^+^ intercalation/deintercalation process under the same testing conditions. At high rates, capacity fading is typically dominated by kinetic and transport limitations; therefore, high capacity retention (i.e., higher than 5C) suggests that Mg doping effectively mitigates charge–transfer limitations and lowers overpotential losses during rapid cycling.

## 4 Conclusions

Metal glycerolates, synthesized via a solvothermal method using metal nitrate salts and glycerol, were successfully employed as hybrid organic–inorganic precursors for the preparation of NCM materials. The investigated compositional range, spanning from low‐ to high‐Ni content, enabled a direct correlation between Ni content, structural disorder, and electrochemical performance. The synthetic approach proved effective for low‐Ni NCM compositions, yielding enhanced structural ordering and electrochemical stability. However, increasing Ni content led to progressive structural disorder and electrochemical degradation, indicating challenges in obtaining high‐performance Ni‐rich layered oxides under the synthetic conditions investigated. Furthermore, Mg doping proved to be an effective strategy to enhance the electrochemical performance of NCM 333, significantly improving both rate capability and cycling stability. The correlation between the structural characterization and electrochemical performance indicates that the increased Li^+^/Ni^2+^ cation mixing was a major contributing factor to the lower discharge capacity of NCM samples with higher Ni content, while the enhanced cycling stability of Mg‐doped NCM 333 is consistent with structural stabilization effects associated with Mg^2+^ incorporation rather than with changes in the Ni oxidation state or a reduction in Li^+^/Ni^2+^ cation mixing. Thus, metal glycerolates emerge as promising precursors for LIB cathodes, providing valuable insights into structure–property relationships and offering a basis for the rational development of cost‐effective and more sustainable energy storage materials.

## Author Contributions

Conceptualization and methodology: Gustavo Thalmer M. Silva and Josué M. Gonçalves. Investigation: Gustavo Thalmer M. Silva and Syra Mubarac. Formal analysis: Gustavo Thalmer M. Silva, Irlan S. Lima, Bruno Freitas, and João Pedro A. dos Santos. Resources: Josué M. Gonçalves and Hudson Zanin. Writing‐Original Draft Preparation: Gustavo Thalmer M. Silva. Writing‐Review and Editing: Gustavo Thalmer M. Silva, Irlan S. Lima, Bruno Freitas, João Pedro A. dos Santos, Josué M. Gonçalves, and Hudson Zanin. Visualization: Gustavo Thalmer M. Silva. Supervision: Josué M. Gonçalves and Hudson Zanin. Funding Acquisition: Josué M. Gonçalves, and Hudson Zanin. All authors have read and agreed to the published version of the manuscript.

## Funding

This work was supported by the Shell (6176.2), the Fundação de Amparo à Pesquisa do Estado de São Paulo (2023/17560‐0, 2025/13375‐0, 2025/03786‐2, 2017/11958‐1, 2017/11986‐5, 2022/04127‐4, and 2014/02163‐7), the Conselho Nacional de Desenvolvimento Científico e Tecnológico (405643/2022‐5 and 302180/2022‐2), and Fundo Mackenzie de Pesquisa e Inovação (#231020 and #251035).

## Conflicts of Interest

The authors declare no conflicts of interest.

## Supporting information

Supplementary Material

## Data Availability

The data presented in this study are available on request from the corresponding authors.
