# Supplementary material for: Metal Glycerolate‐Based Synthesis and Structure–Property Relationships of LiNi1−x−y Co x Mn y O2 Cathodes for Lithium‐Ion Batteries
Source: ChemistryOpen. 2026 Oct 28;15(11):e70327. doi: 10.1002/open.70327 (PMC13716698; doi:10.1002/open.70327)
Supplement: Supplementary file 1 [file OPEN-15-e70327-s001.pdf]

Supporting information for:

Metal glycerolate-based synthesis and structure-property relationships of $LiNi_{1-x-y}Co_xMn_yO_2$ cathodes for lithium-ion batteries

Gustavo Thalmer M. Silva,*[1,2,3] , Syra Mubarac,[3] Irlan S. Lima,[4] Bruno Freitas,[3] João Pedro A. dos Santos,[3] Josué M. Gonçalves*[2,5], Hudson Zanin*[3]

[1]Center for Studies in Energy and Oil, University of Campinas, Rua Cora Coralina 350, Campinas, SP, 13083-896, Brazil

[2]Mackenzie Institute for Research in Graphene and Nanotechnologies (MackGraphe), Mackenzie Presbyterian Institute, Rua da Consolação 930, São Paulo, SP, 01302-907, Brazil.

[3]Advanced Energy Storage Division, Center for Innovation on New Energies, School of Electrical and Computer Engineering, University of Campinas, Av. Albert Einstein 400, Campinas, SP 13083-852, Brazil.

[4]Department of Fundamental Chemistry, Institute of Chemistry, University of São Paulo, Av. Prof. Lineu Prestes, 748, 05508-000, São Paulo, SP, Brazil

[5]Mackenzie School of Engineering, Mackenzie Presbyterian University, Rua da Consolação 930, São Paulo, SP, 01302-907, Brazil

* Corresponding author
E-mail address: thalmer@usp.br; josue.goncalves@mackenzie.br and hzanin@unicamp.br

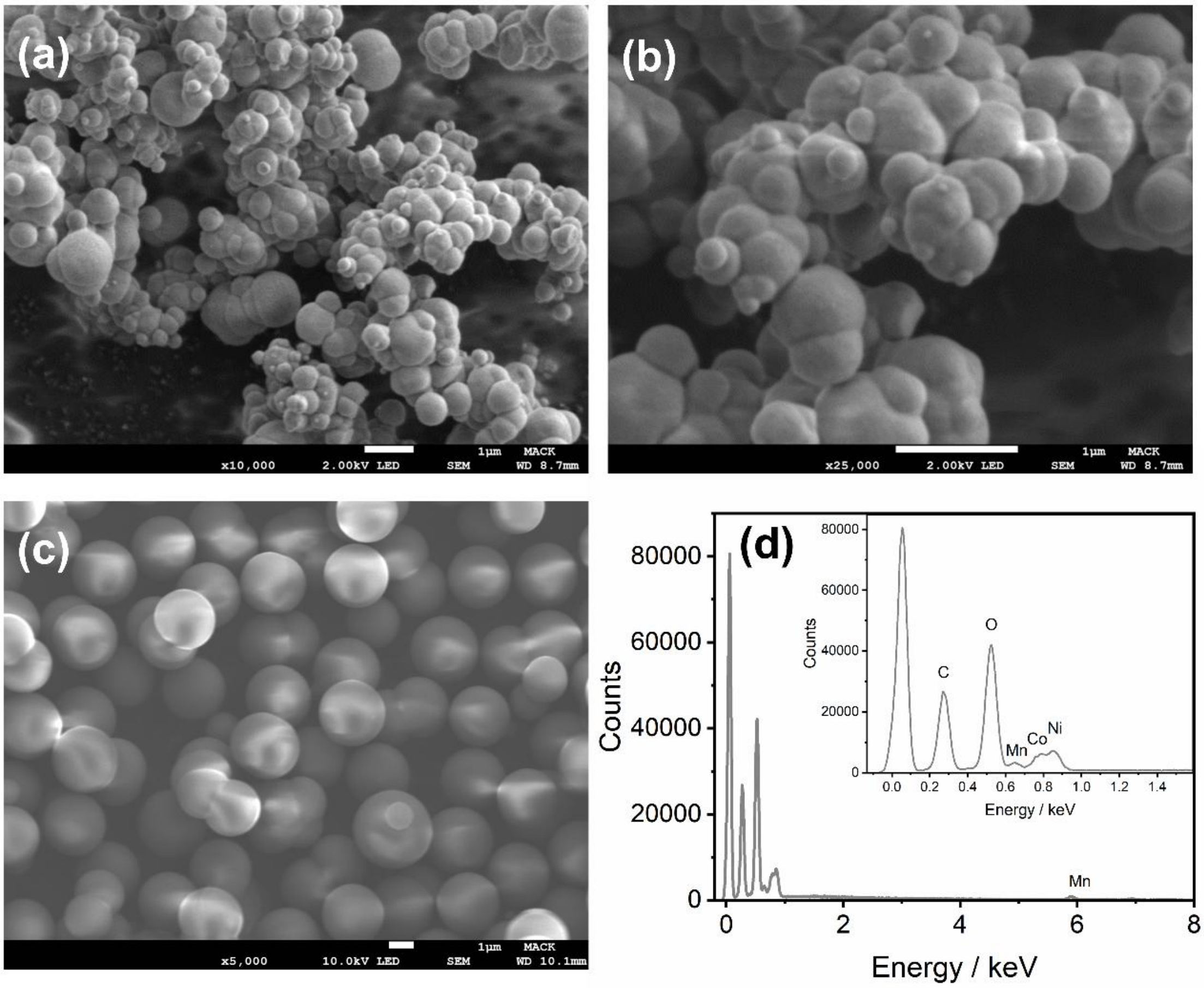

Figure S1 - SEM images of (a-b) M-glycerolate (100% Ni) and (c) M-glycerolate (Ni:Co:Mn = 1:1:1); (d) SEM-EDS mapping for M-glycerolate (Ni:Co:Mn = 1:1:1).

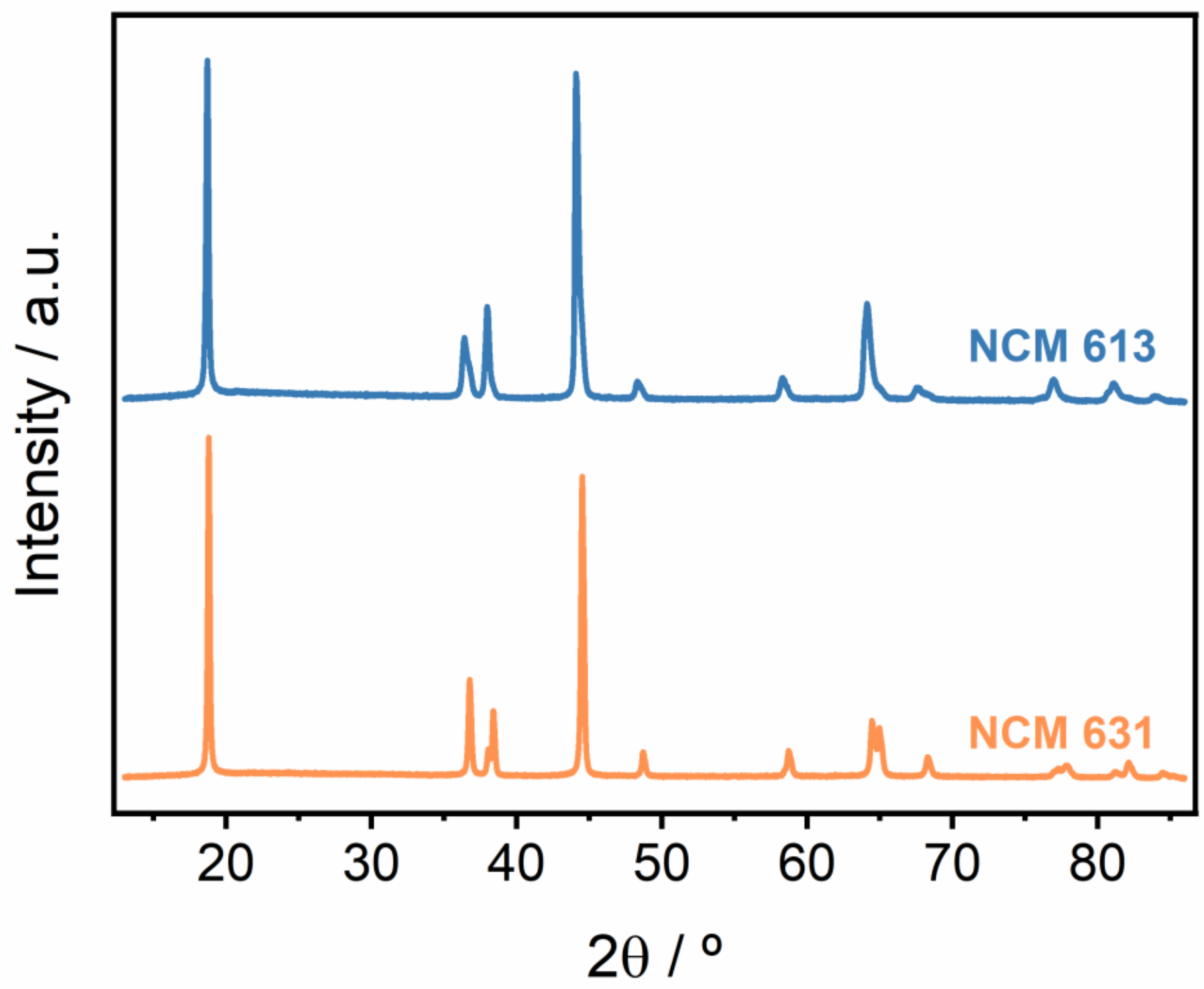

Figure S2 - XRD patterns of NCM 631 and NCM 613.

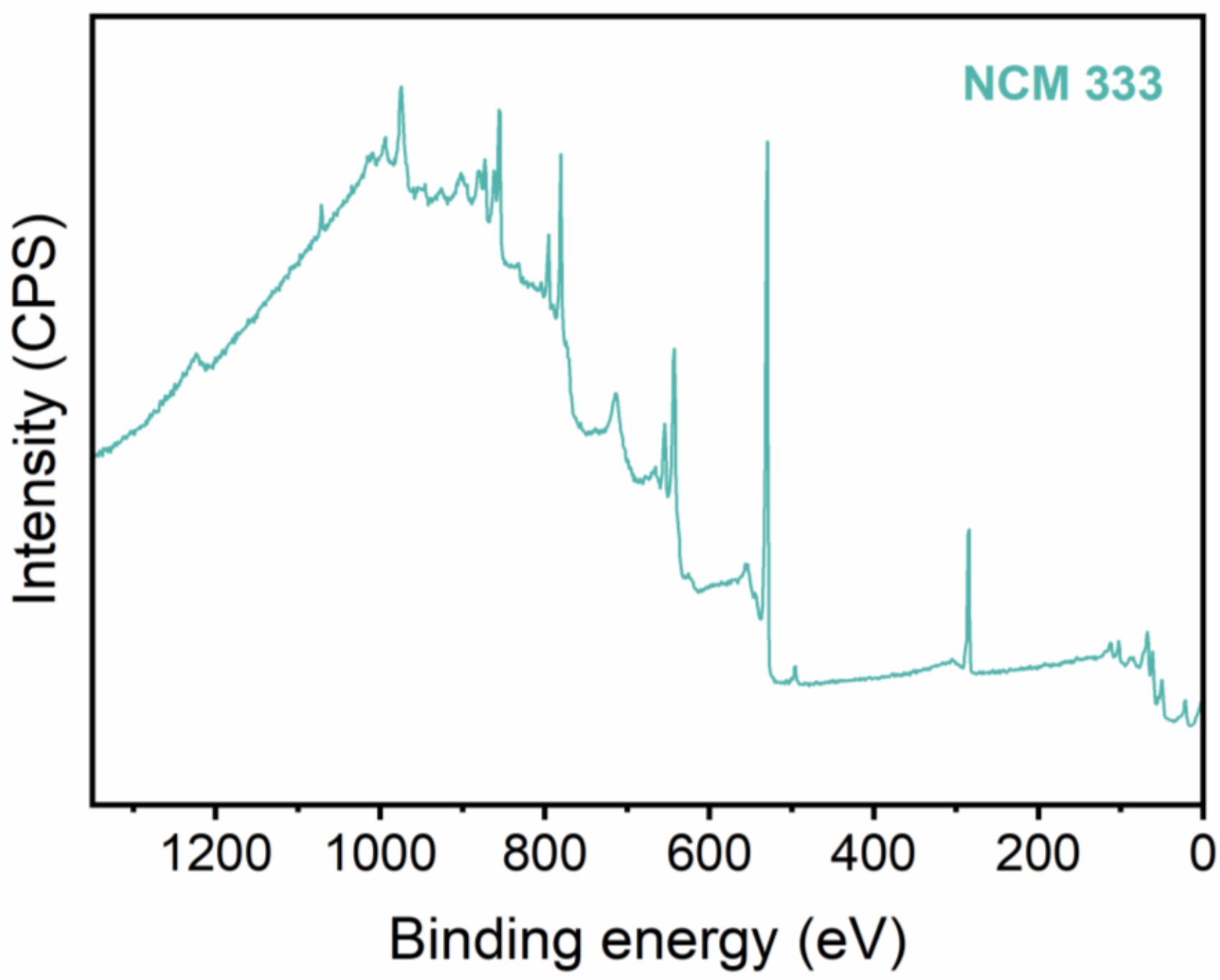

Figure S3 – Survey XPS spectrum of NCM 333.

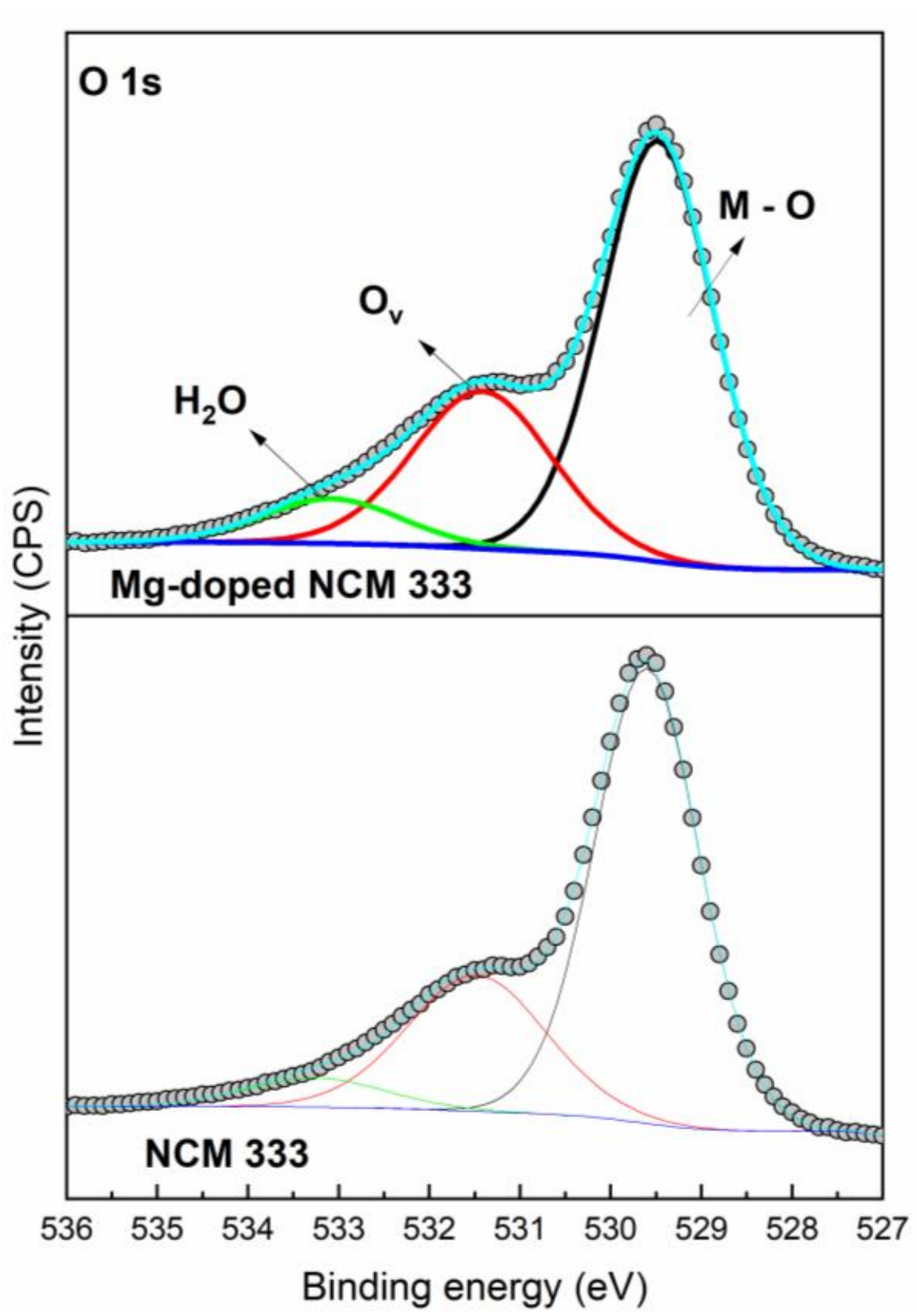

Figure S4 – O 1s XPS spectra for NCM 333 and Mg-doped NCM 333.

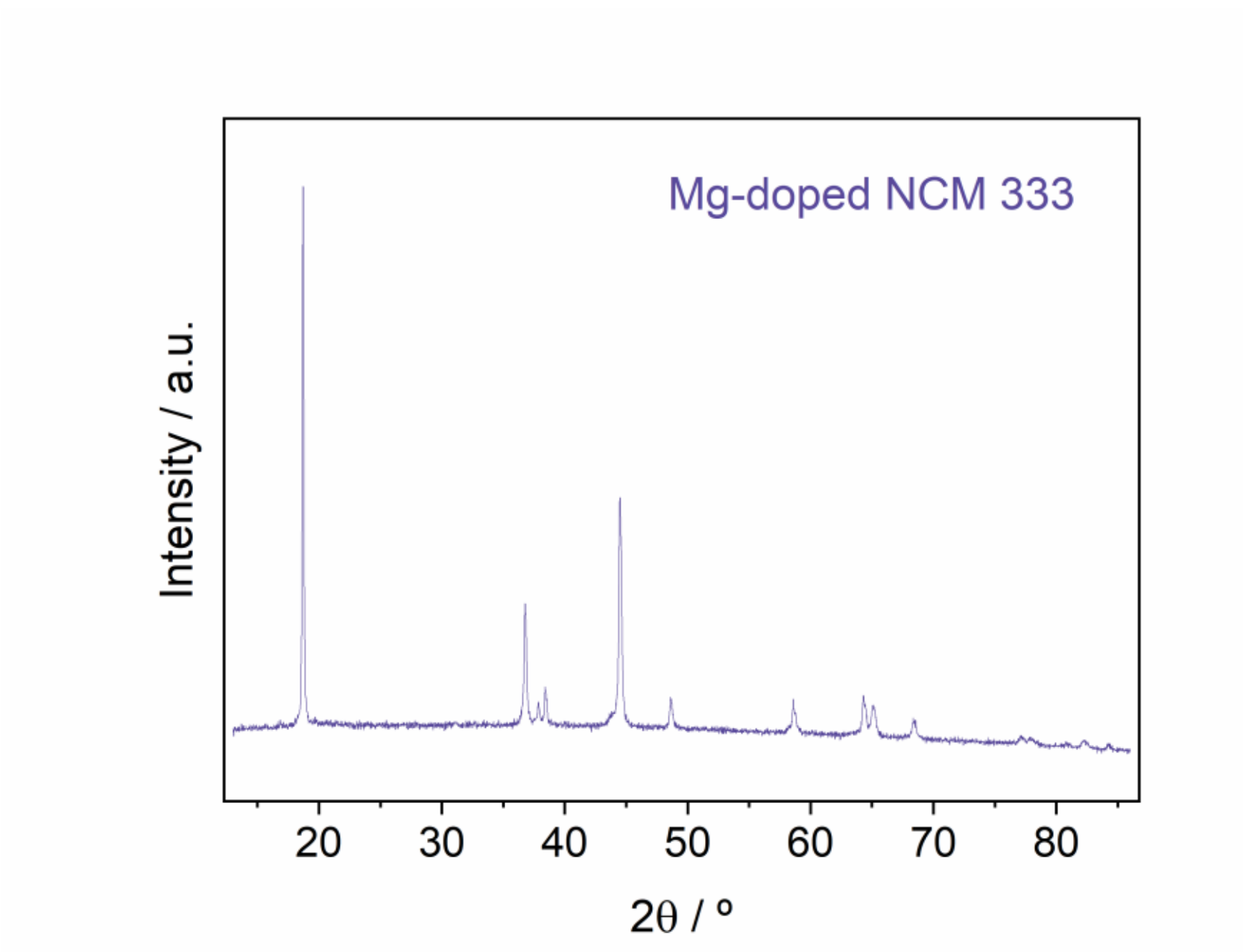

Figure S5 – XRD pattern of Mg-doped NCM 333.

Table S1. Integrated cathodic area and apparent CV-derived discharge capacity of NCM 333 and Mg-doped NCM 333 at different scan rates.

Scan rate (mV s^{-1})	Cathodic area NCM 333 (mA V)	Q_{dis} NCM 333 (mAh g^{-1})	Cathodic area Mg-doped (mA V)	Q_{dis} Mg-doped (mAh g^{-1})	Capacity increase (%)
0.1	0.358	111.43	0.417	125.29	12.4
0.2	0.677	105.28	0.811	121.73	15.6
0.3	0.976	101.24	1.163	116.45	15.0
0.4	1.256	97.66	1.492	112.01	14.7
0.5	1.496	93.09	1.809	108.64	16.7
1.0	2.541	79.06	3.224	96.85	22.5

Table S2. Anodic and cathodic peak currents extracted from the CV curves of NCM 333 and Mg-doped NCM 333 at different scan rates.

Scan rate (mV s^{-1})	Anodic peak current, i_{pa} (A g^{-1}) NCM 333	Cathodic peak current, i_{pc} (A g^{-1}) NCM 333	Anodic peak current, i_{pa} (A g^{-1}) Mg-doped NCM 333	Cathodic peak current, i_{pc} (A g^{-1}) Mg-doped NCM 333
0.1	0.162061	-0.081547	0.168329	-0.096311
0.2	0.269411	-0.133229	0.297547	-0.171307
0.3	0.383228	-0.182134	0.425767	-0.232661
0.4	0.486031	-0.219312	0.527624	-0.285483
0.5	0.581002	-0.247639	0.623438	-0.340671
1.0	0.895819	-0.396086	1.050592	-0.553378
